# Assessment of the educational sensory–based approach for dental treatment of children with autism in Central Italy

**DOI:** 10.3389/froh.2025.1731639

**Published:** 2026-01-20

**Authors:** Denise Corridore, Mario Trottini, Gianni Di Giorgio, Giulia Zumbo, Ida Carmen Corvino, Alessandro Salucci, Matteo Nagni, Iole Vozza, Maurizio Bossù

**Affiliations:** 1 Department of Life Science, Health, and Health Professions, Link Campus University Via del Casale di San Pio V, Rome, Italy; 2 Department of Mathematics, University of Alicante, Alicante, Spain; 3 Department of Oral and Maxillofacial Sciences, Sapienza University of Rome, Rome, Italy; 4 Dental School, IRCCS San Raffaele Hospital, Vita-Salute San Raffaele University, Milan, Italy

**Keywords:** autism spectrum disorder, dental anxiety, health education, oral health management, patients with disabilities

## Abstract

**Background:**

For some children with autism spectrum disorder (ASD), over-responsivity to sensory stimuli in a dental office environment and communication barriers can result in uncooperative behavior, in extreme cases necessitating the use of general anesthesia. Tailored educational approaches are a promising tool to address these issues.

**Objective:**

This study assesses the effectiveness of an existing educational approach, called the educational sensory–based approach (ESBA), which aims to improve cooperation during dental care treatment of children with ASD. The relevant research questions are whether children improve their levels of cooperation during the implementation of the phases of the ESBA and how such improvement depends on study variables. According to our definition, an initially uncooperative child (Frankl scale at first visit rated *negative* or *definitely negative*) is considered to have *improved* by the end of a certain phase if their Frankl scale rating at the end of the phase is *positive* or *definitely positive*, while an initially cooperative child (Frankl scale at first visit rated *positive*) is considered to have *improved* by the end of a certain phase if their Frankl scale rating at the end of the phase is *definitely positive*.

**Methods:**

In this study, a retrospective repeated-measures design was used. The final sample comprised 45 initially uncooperative and 40 initially cooperative children with ASD who completed the ESBA program between 2013 and 2020. Data included demographic and clinical examination variables, medical history, and child behavior and cooperation. A statistical analysis was performed using 3,328 cumulative logit models to address the relevant research questions.

**Results:**

A statistically significant improvement across the different phases of the ESBA program was observed, independent of the other explanatory variables in the study. The 95% confidence intervals for the predicted probability that an initially uncooperative child would *improve* by the end of the ESBA program were [0.71 and 0.88], whereas the probabilities for an initially cooperative child *improving* were lower at [0.04 and 0.20].

**Conclusions:**

The ESBA represents a promising tool for managing dental care in children with ASD. It facilitates cooperation and limits reliance on general anesthesia. The findings from this study can inform clinical practice in pediatric dentistry, particularly in managing patients with ASD, and provide a starting point for other medical teams to implant and implement alternative educational approaches.

## 1 Introduction

Autism spectrum disorder (ASD) is a developmental disorder characterized by deficits in social communication, repetitive behaviors, and restricted interests. Diagnostic criteria have evolved over time, with the current standards defined in both the *Diagnostic and Statistical Manual of Mental Disorders, Fifth Edition* (DSM-5) and the *International Classification of Diseases, Eleventh Revision* (ICD-11), which provide harmonized frameworks for identifying ASD based on symptom presentation and functional impact. In DSM-5, the condition is accompanied by three levels of support need (from “requiring support” to “requiring very substantial support”), which indicates the extent to which symptoms interfere with daily functioning. ICD-11 maintains the spectrum concept but classifies ASD according to intellectual functioning and the presence of functional language, generating four subtypes that reflect cognitive–linguistic profiles. Additional specifiers allow clinicians to identify associated medical, genetic, or neurodevelopmental conditions. Taken together, DSM-5 focuses on graded support requirements, whereas ICD-11 emphasizes cognitive and language characteristics, offering complementary frameworks for clinical evaluation and research (1–7).

Interventions for people with ASD need to be accompanied by broader actions for making physical and social environments more accessible, inclusive, and supportive for them. This is especially important in the context of dental care and dental treatment. For some autistic children, normal daily activities such as tooth brushing and understanding the behavior of other individuals constitute a constant challenge. In addition, compared with typically developing children, dental anxiety (that may be induced by the novel environment and stressor stimuli present in dental practice) is more common in children with ASD (and, in general, in children with neurodevelopment disorders) as a result of the greater prevalence of sensory overresponsivity in these populations (7–10). This may result in uncooperative behaviors such as crying or physical and verbal aggression so as to avoid treatment. In children with ASD, it is often more difficult to interpret the signs of fear and anxiety in advance, making it harder to prevent the loss of cooperation. The results of this lack of cooperation are a significantly larger number of untreated caries, an increased prevalence of periodontitis and gingivitis, and a high prevalence of treatment under general anesthesia compared with the average population (11–13).

Several studies suggest that tailored educational and behavioral approaches can enhance cooperation, reduce anxiety, and improve the overall safety and effectiveness of dental treatment in children with ASD. For example, one study (10) reported that anxiety modulation techniques, graduated exposure, and consistent behavioral frameworks were essential for managing uncooperative behaviors in this population. Similarly, another study (14) discussed the importance of adapting the clinical environment and adopting predictable, patient-centered behavioral protocols in dental treatment of children with ASD. More recent studies provided evidence that multicomponent educational programs and interdisciplinary behavioral management significantly improved the ability of patients to tolerate routine procedures (15) and emphasized the value of structured preparatory interventions and the integration of technological aids to reduce sensory overload and facilitate communication in children with ASD (16).

Inspired by these ideas, the Dentistry Unit at Policlinico Umberto I (Rome) began, in 2008, to develop a new behavioral approach for children with ASD, implementing an “educational sensory-based” method customized to each patient. This approach, called the educational sensory–based approach (ESBA), is described in detail in ref. (17). This approach positions education as a tool for perfecting dental treatment by limiting traumatic experiences to the patient, allowing continuity of procedures, and thereby improving cooperation while reducing the use of general anesthesia (18–21).

The goal of this paper is to evaluate the efficacy of the ESBA using data collected from children aged 7–12 years who completed their ESBA program in the Unit of Pediatric Dentistry at Policlinico Umberto I.

## 2 Materials and methods

For this study, a retrospective repeated-measures design was used. Eligible participants were children aged between 7 and 12 years, diagnosed with ASD by a qualified public health professional (according to the ICD-11 criteria), who had started and completed the ESBA program at the Department of Oral and Maxillo-Facial Science, Unit of Pediatric Dentistry and Special Needs Patients, Policlinico Umberto I in Rome, during the period between June 2013 and March 2020. Children were excluded if they met any of the following exclusion criteria:

- Suffered from other diseases known to influence dental caries or the severity of periodontal disease, such as Down's syndrome and diabetes.
- Lacked parental consent to participate in the study (for each participant, an informed consent form was required to be signed by the parents).

The final sample included 85 children. Data were collected during the ESBA program. Parents or legal guardians provided informed consent for the use of recorded data for research purposes. The study was conducted in accordance with the principles of the Declaration of Helsinki and was formally reviewed and approved by the Board of the Department of Oral and Maxillo-Facial Sciences, Pediatric Dentistry Unit, Policlinico Umberto I, Rome, Italy (Approval No. 15/2020, Prot. No. 0000216, dated 6 February 2020). Although the ESBA approach was conceptualized in 2008, during the period between March 2008 and March 2013, the approach underwent several modifications based on a trial-and-error procedure before reaching its final version, and it was implemented from June 2013. This was why the observations collected between March 2008 and March 2013 were excluded from this study. Data collected after March 2020 were also excluded because of the COVID-19 pandemic.

### 2.1 Data collection

Prior to the start of the ESBA program, the authors DC, GD, GZ, and IC (dentists and hygienists of the Unit of Pediatric Dentistry and Special Needs Patients, Policlinico Umberto I) completed a 1-year training period with a licensed therapist, with formal training and certified experience in autism spectrum disorders (specifically in behavioral, relational, and communication strategies used in evidence-based interventions), to acquire the relational skills necessary for effective communication and interaction with the children participating in the program. Skill acquisition was ensured through supervised training, direct observation, and competency-based evaluation during the 1-year period. The dentists and hygienists involved in the program were required to demonstrate adequate proficiency in the use of communication strategies, behavior support techniques, and child–clinician interaction methods before participating in the program.

In preparation for clinical activities, intra- and inter-examiner reliabilities were assessed to ensure consistency in caries detection and oral health status evaluation. Calibration was performed on a sample of pediatric patients not involved in the study, following the World Health Organization guidelines for oral health surveys. The assessment was conducted on a tooth-by-tooth basis using Kappa statistics (22). Kappa values of 0.82 (for intraexaminer) and 0.84 (for interexaminer) were obtained.

For each of the 85 children in the final sample, a clinical history was retrospectively obtained from the patient's’ clinical charts that the dentist completed at the end of each visit during the period of study (June 2013–March 2020). In this respect, it should be noted that in the ESBA program, treatments followed a structured sequence of weekly sessions over three main phases and a follow-up session, as described in Table 1. Throughout all phases, a licensed autism therapist monitored the sessions, provided feedback to the medical team, and suggested strategies for improvement. When a lack of cooperation prevented a certain treatment to be given and the treatment required urgent implementation (e.g., trauma, pain, and rapid progression of disease or infection), the child was referred for treatment under general anesthesia.

**Table 1:** Description of ESBA phases.

Phase	Number of sessions	Aims/tasks
Phase I	4–6 sessions	Establishes the doctor–patient relationship, builds routine, and may include initial preventive or operative care
Phase II	3–4 sessions	Consolidates the relationship and continues preventive and/or operative treatments
Phase III	1–2 sessions	Focuses on maintaining cooperation and motivation
Follow-up	1–2 visits every 4 months	Includes 1–2 visits every 4 months for hygiene, prevention, or further treatments depending on the patient

The clinical charts, used to obtain the clinical history of the children in the study, contained information about the treatments that the dentist had completed during each visit, as well as the dentist's annotations about both positive and negative child behaviors (e.g., acceptance, reluctance to accept or refusal of the treatments, crying, willingness to comply with the dentist, negative attitudes, and fear).

Based on the clinical history of the 85 children in the study, the dentists retrospectively rated each child’s behavior and cooperation, assigning (for the first visit and for the end of each of the three phases in the study) a value corresponding to one of the four categories of the Frankl scale (*definitely negative*, *negative*, *positive*, and *definitely positive*). The Frankl scale is a widely used and reliable behavior rating system in both clinical dentistry and ASD research (18, 23, 24).

From the clinical history, an additional outcome variable, *Type of treatment*, was retrospectively assessed at the end of each of the three phases established in the ESBA. For a given child, the variable *Type of treatment* represents the type of treatments received by the child during a particular phase according to the following classifications: approach, checkups, preventive procedures, dental cleaning, conservative procedures, surgical procedures, and anesthesia. It must be noted that during a certain phase, a patient can undergo more than one treatment.

From the participants' records at the first visit, 10 baseline covariates were also retrospectively obtained. These covariates were related to demographic attributes (age at the first visit, gender, nationality), medical history (type of delivery, vaccines, medications, previous experiences of general anesthesia), and clinical data (dental health status, gingival status, observational plaque index).

Information on demographic attributes and medical history was obtained from a questionnaire, especially designed for the study, completed by parents in a face-to-face interview with the dentist at the first visit. Clinical data were obtained from the records of the initial examination performed at the Unit of Pediatric Dentistry and Special Needs Patients, Policlinico Umberto I.

The recorded values of the sum of decayed, missing, and filled teeth in the primary dentition (dmft) and in the permanent dentition (DMFT), denoted by DMFT/dmft, were used to determine the dental health status. The recorded values of plaque and gingival indexes (with four and three categories, respectively, described in Table 2) were used to assess the general prevalence of periodontal health in accordance with the World Oral Health Survey (WHO) guidelines (25, 26).

**Table 2:** Summary statistics for the variables in the study related to demographic attributes, medical history, and clinical data.

Variable	Cohort of children		Variable	Cohort of children	
	Uncooperative at first visit	Cooperative at first visit		Uncooperative at first visit	Cooperative at first visit
Gender	(45)	(40)	Natural birth	(41)	(35)
Male	68.9%	77.5%	Yes	39.0%	48.6.5%
Female	31.1%	22.5%	No	61.0%	51.4%
Vaccination	(38)	(35)	Medication	(42)	(38)
Yes	92.1%	88.6%	Yes	21.4%	18.4%
No	7.9%	11.4%	No	78.6%	81.6%
Nationality	(45)	(40)	Previous anesthesia	(45)	(39)
Italian	89.9%	100%	Yes	11.1%	15.4%
Other	11.1%	0%	No	89.9%	84.6%
Plaque index	(45)	(40)	Gingival status	(45)	(40)
Absence of plaque	4.4%	12.5%	Healthy	8.9%	20.0%
Detectable plaque	24.4%	30.0%	Bleeding	33.3%	32.5%
Visible plaque	53.3%	40.0%	Tartar	57.8%	47.5%
Abundant accumulation	17.8%	17.5%			
Age	(45)	(40)	Dental Health Status DMFT/dmft	(45)	(40)
Min.	7.0	7.0	Min.	0.0	0.0
First quartile	8.0	8.8	First quartile	0.0	0.0
Median	9.0	9.0	Median	2.0	2.5
Mean	9.2	9.4	Mean	3.1	3.1
Third quartile	10.0	11.0	Third quartile	5.0	5.0
Max.	12.0	12.0	Max.	11.0	10.0
SD	1.51	1.53	SD	2.9	3.0

Sample size is reported in parenthesis.

### 2.2 Statistical analysis

As a first step in the data analysis, univariate distributions and summary statistics for the variables in the study were computed separately for the two subcohorts of children defined by their uncooperative and cooperative behaviors at the initial visit. The first cohort (children with initially uncooperative behavior) comprised the 45 children in the sample with the Frankl scale rating of *negative* or *definitely negative* at the first visit*.* The second cohort (children with initially cooperative behavior) comprised the 40 children in the sample with the Frankl scale rating of *positive* at the first visit (none of the children in the sample were rated as *definitely positive* at the first visit).

The distinction between the two groups is important because the effectiveness of the ESBA has a different meaning for the two subcohorts. As the primary goal is successful dental treatment, cooperative behavior is required on the part of children. For initially uncooperative children, we consider the ESBA approach to be successful if, after intervention, the children's behavior changes to cooperative (the Frankl scale changes from *negative* or *definitely negative* to *positive* or *definitely positive* during the program). On the other hand, for initially cooperative children, we consider the ESBA approach to be successful if their cooperation (already present before intervention) improves during the program (the Frankl scale changes from *positive* to *definitely positive* during the program). The underlying rationale is that, for initially cooperative children, improvement in cooperation reflects an improved perception of the dental experience, which, in turn, may simplify and accelerate the administration of the required treatments and reduce distress.

In order to distinguish between the two cohorts of children and measure the success of the ESBA approach, we defined the new variable, *Init.Rating*, and the notion of *improvement*. The *Init.Rating* variable takes the value 0 for children with initially uncooperative behavior (with the Frankl scale rating of *negative* or *definitely negative* at the first visit) and the value 1 for children with initially cooperative behavior (with the Frankl scale rating of *positive* at the first visit). The notion of *improvement* takes into account the question whether the ESBA approach has achieved its goals by the end of a given phase: (i) A child demonstrating initially uncooperative behavior (*Init.Rating* = 0) is considered to have *improved* by the end of phase *k* (*k* = I, II, III) if their Frankl scale rating is either *positive* or *definitely positive*. (ii) A child demonstrating initially cooperative behavior (*Init.Rating* = 1) is considered to have *improved* by the end of phase *k* (*k* = I, II, III) if their Frankl scale rating is *definitely positive*.

As a preliminary assessment of the efficacy of the ESBA, for each of the two cohorts of initially uncooperative and cooperative children, we computed the distribution of the type of treatment performed and the percentage of children who had *improved* by the end of phases I, II, and III. In order to test the statistical significance of the differences between the Frankl scale ratings at baseline (i.e., at the first visit) and the corresponding rate at the end of each of the three phases, the Stuart–Maxwell test (27) was used.

A more rigorous assessment of the efficacy of the ESBA was conducted through statistical modeling, relating the response variable (Frankl scale assessment) to the set of explanatory variables in the study. A categorical predictor with *m* levels was represented using *m−1* dummy variables. In all cases, the reference category was the first level of the variable, as reported in Table 2.

In order to exploit the ordering of the components of the response variable (Frankl scale assessment is an ordinal categorical variable with four levels), an analysis was performed by fitting a set of 3,328 cumulative logit models with proportional odds, the most widely used logistic model for ordinal response (27). Each of the 3,328 models corresponded to a different set of explanatory variables and interactions to be included in the modeling. All models were fitted using the R software (version 4.3.1) and the *clm()* function from the *Ordinal* package available from the Comprehensive R Archive Network (CRAN) at https://CRAN.R-project.org/package=ordinal (28).

Model selection was performed using the Bayesian Information Criterion (BIC), which is a penalized likelihood method that favors parsimonious models and has a Bayesian interpretation in terms of the posterior probability of the models (29). The best model is the one with the minimum BIC.

In order to account for uncertainty in BIC scores due to sampling variability, the “one standard error rule” (*1se rule*) for model selection was applied. The rule was first proposed in (30) for tree size selection. In our study, the rule was implemented in three steps. Step 1: 2,000 bootstrap “copies” of the original dataset were produced. Step 2: For each bootstrap sample, the 3,328 cumulative logit models were refitted, and the corresponding BIC score were computed. In this way, for each model, 2,000 bootstrap BIC scores were produced, for which the average and the standard deviation were computed. Step 3: The model with the minimum average BIC was identified. Let $\bar{BIC}$ and *se* be the average and the standard deviation of the 2,000 BIC scores for this model, respectively. The *best BIC-1se model* was defined as the most parsimonious model whose average BIC fell within one standard deviation of $\bar{BIC}$ [i.e., within the interval ( $\bar{BIC}$ *-se,* $\bar{BIC}$ *+se*)].

The *best BIC-1se model* was then used to compute 95% confidence intervals for the probability that an initially uncooperative child and an initially cooperative child would *improve* by the end of phase I, II, and III of the ESBA. The 95% confidence intervals were obtained using the bootstrap and the percentile method (31). Again, 2,000 bootstrap “copies” of the original dataset were produced. For each bootstrap sample, the selected BIC-1se model was refitted and the corresponding predicted probabilities of interest were computed. In this way, for each event of interest, 2,000 predicted bootstrap probabilities were produced. The corresponding 95% confidence interval was obtained by computing the 0.025 and 0.975 percentiles of these 2,000 predicted bootstrap probabilities.

## 3 Results

### 3.1 Descriptive findings

Summary statistics for the variables in the study are presented in Tables 2–4, separately for the two cohorts of children with uncooperative and cooperative behaviors at the initial visit (45 and 40 children, respectively). For each variable, the actual sample size (*n*) is reported in parentheses.

**Table 3:** Percentage of children who received a given treatment by the end of phase I, phase II, and phase III for the two subcohorts of children with uncooperative and cooperative behaviors at the first visit.

Type of treatment	Cohort of children					
	Initial uncooperative (*n* = 45)			Initial cooperative (*n* = 40)		
	Up to phase I	Up to phase II	Up to phase III	Up to phase I	Up to phase II	Up to phase III
Education intervention	95.6%	97.8%	100%	77.5%	77.5%	82.5%
Checkups	62.2%	64.4%	66.7%	0%	0%	10.0%
Preventive procedures	0%	46.7%	64.4%	47.5%	70%	77.5%
Dental cleaning	33.0%	53.3%	66.7%	57.5%	67.5%	87.5%
Conservative procedures	2.2%	11.1%	15.6%	57.5%	70%	72.5%
Surgical Procedures	0%	4.4%	11.1%	17.5%	22.5%	22.5%
Anesthesia	11.1%	26.7%	26.7%	0%	10.0%	10.0%

**Table 4:** Distribution of Frankl scale ratings at the first visit and at the end of phase I, phase II, and phase III for the two subcohorts of children with uncooperative and cooperative behaviors at the first visit.

Time of assessment	Frankl scale rating for initial uncooperative children (*n* = 45)				Frankl scale rating for initial cooperative children (*n* = 40)			
	− −	−	+	++	− −	−	+	++
First visit	13.3%	86.7%	0	0	0	0	100%	0
End of phase I	11.1%	53.3%	**35**.**6%**	0	0	0	97.5%	**2**.**5%**
End of phase II	2.2%	37.8%	**57**.**8%**	**2.2%**	0	10%	87.5%	**2**.**5%**
End of phase III	0%	15.6%	**80**.**0%**	**4.4%**	0	2.5%	90%	**7**.**5%**

Boldface is used to denote cases in which the patients *improved* with respect to the first visit. According to our definition, a child initially uncooperative, i.e., with a Frankl scale rating of *negative* (−) or *definitely negative* (–) at first visit, has improved by the end of a certain phase if their rating at the end of the phase is *positive* (+) or *definitely positive* (++); a child initially cooperative, i.e., with a Frankl scale rating of *positive* (+) at first visit, has improved by the end of a certain phase if their rating at the end of the phase is *definitely positive* (++).

The univariate distribution of the variables related to demographic attributes (age at the first visit, gender, nationality), medical history (type of delivery, vaccines, medications, previous experiences of general anesthesia), and clinical data (dental health status, gingival status, observational plaque index) is presented in Table 2. The first two groups of variables display similar distribution patterns in the two cohorts of children with uncooperative and cooperative behaviors at the initial visit. In both cohorts, approximately three-quarters of the sample are boys and the age of the children ranges between 7 and 12 years, with a mean of approximately 9.2 and a standard deviation of 1.5. The vast majority of the children (more than 88.6% in both cohorts,) are Italian and received vaccinations on schedule according to the Italian National Health Service Plan. Fewer than half of the children were born by natural childbirth. With regard to medication use, based on parental/legal guardian declarations collected through questionnaires, approximately 20% of the children in both cohorts regularly or temporarily used medications, either for anxiety and tension control or for comorbidity with pathologies of a different origin (cardiac, neurological, or psychiatric). Previous experience with anesthesia was reported in 15.4% of children with cooperative behavior at the first visit and 11.1% of children with initially uncooperative behavior. In the latter group, the large majority (91.1%) presented with bleeding and/or gingival tartar, and approximately seven out of 10 (71.1%) had visible plaque at the gingival margin or abundant accumulation of calculus beyond the margin. These percentages dropped to 80.0 and 57.5, respectively, in the cohort of children with cooperative behavior at the first visit. The average DMFT/dmft index was 3.1 in both cohorts, with a standard deviation of approximately 2.9.

As a preliminary descriptive evidence of the efficacy of the ESBA, Table 3 reports, for each level (treatment) of the variable *Type of treatment*, the percentage of patients in the two cohorts who had received the treatment during phase I, by the end of phase II, and by the end of phase III. In phase I, educational intervention was carried out in the vast majority (95.6%) of the 45 initially uncooperative children at the first visit and in the majority (77.5%) of the 40 initially cooperative children at the first visit. These percentages had increased to 100 and 82.5, respectively, by the end of phase III. Regular checkups were performed in approximately 60% of the initially uncooperative children (in 62.2% of them in phase I and in 64.4% and 66.7% by the end of phase II and III, respectively), whereas they were largely postponed until phase III for the initially cooperative children (10% of these children received a checkup in phase III and none of them received a checkup in the first two phases of the ESBA). A substantial percentage of the initially cooperative children received preventive procedures (such as routine dental cleaning and fluoride treatments) (47.5) and more demanding/invasive treatments such as conservative procedures (57.5), minor surgery (17.5), and dental cleaning (57.5). This, however, was not the case for the cohort of initially uncooperative children; in phase I, only 33.3% of this cohort received dental cleaning and less than 2% received preventive or conservative procedures and minor surgeries. As children progressed through phases II and III of the ESBA program, some of these differences between the two cohorts narrowed, while others largely persisted. By the end of phase III, 66.7% of the initially uncooperative children at the first visit had received dental cleaning and approximately the same percentage (64.4) preventive procedures (remarkable increases compared with phase I, where these percentages were 33.3 and 0, respectively). The corresponding percentages for the initially cooperative children—who by the end of phase III had received dental cleaning and preventive procedures—were not too different (87.5 and 77.5, respectively).

The percentages of initially uncooperative children who received conservative and surgical procedures also increased significantly during the ESBA program (from 2.2 in phase I to 15.6 by the end of phase III for conservative procedures and from 0 in phase I to 11.1 in phase III for surgical procedures). Despite this favorable evolution, these percentages remained much lower (approximately one-fifth and half) than the corresponding percentages for the cohort of children who were initially cooperative, among whom 72.5% of the children had received conservative procedures and 22.5% surgical procedures by the end of phase III.

In both cohorts, when educational intervention did not translate into collaboration and treatment deferral was not an option (due to the urgency of the case), the patients were treated under general anesthesia. Five (11.1%) of the initially uncooperative children were treated under general anesthesia in phase I and 12 (26.7%) by the end of phase III. These percentages were much lower in the cohort of initially cooperative children. None of the children in this group (0%) was treated under anesthesia in phase I and only four (10%) were treated by the end of phase III. Notably, these four children, who although cooperated at the first visit, demonstrated a decline in cooperation in phase II of the ESBA.

Additional descriptive evidence of the ESBA efficacy is provided in Table 4, which shows the distribution of the Frankl scale at the first visit and at the end of phases I, II, and III for the two cohorts of children with initial uncooperative and cooperative behaviors. Boldface is used to denote cases in which the patients *improved* compared with the first visit. As we explained earlier, according to our definition, an initially uncooperative child (with a Frankl scale rating of *negative* or definitely *negative* at first visit) was considered *improved* if their rating at the end of the phase became *positive* or *definitely positive*, while an initially cooperative child (with a Frankl scale rating of *positive* at first visit) was considered *improved* if their rating at the end of the phase became *definitely positive*.

As shown in the table, at the first visit, six of the 45 initially uncooperative children (13.3%) were rated in the Frankl scale as *definitely negative*, while the remaining 39 (86.7%) were rated as *negative*. However, as they completed the different phases of the ESBA program, most of these children demonstrated cooperation (*improved* according to our definition). By the end of phase II, 60% had displayed cooperative behaviors, i.e., their Frankl scale rating was either *positive* (57.8%) or *definitely positive* (2.2%). This percentage had increased to 84.4 by the end of phase III (in which 80% of the initially uncooperative children were rated as *positive* and 4% as *definitely positive*). None of the initially uncooperative children regressed in their behavior during the ESBA program.

In order to test whether the outlined differences between the distribution of Frankl scale ratings at the first visit and the corresponding distributions at the end of each ESBA phase were statistically significant, we used the Stuart–Maxwell test. The *p*-values for this test (one for each phase) were all lower than 0.001, indicating that for the cohort of 45 initially uncooperative children, these differences were statistically significant at the *α* = 0.05 level.

If a numerical score from 1 to 4 is assigned to the four levels of the Frankl scale (1 corresponding to *definitely negative* and 4 to *definitely positive*), the sample average rating for these 45 children at the first visit will increase from 1.9 to 2.2, 2.6, and 2.9 by the end of phases I, II, and III, respectively, indicating an upward trend from negative to positive behavior.

On the other hand, as shown in Table 4, the 40 initially cooperative children (all rated as *positive* at the first visit) displayed very limited change in the Frankl scale ratings from the first visit through the phases of the ESBA program. The percentage of these children who maintained the same rating as at the first visit was 97.5 at the end of phase I, 87.5 at the end of phase II, and 90 at the end of phase III. Only one child (2.5% of the total) had *improved* (i.e., their rating had changed to *definitely positive*) by the end of phase II, and this percentage had increased only to 7.5 (i.e., three children) by the end of phase III. In addition, four children (10%) with initially cooperative behaviors regressed in phase II (they changed their behavior to uncooperative and were rated as *negative* in the Frankl scale at the end of phase II). While three of these children returned to their original rating (*positive*) during the last phase of the ESBA program, one maintained the *negative* rating also in phase III. If, as before, a numerical score from 1 to 4 is assigned to the four levels of the Frankl scale (1 corresponding to *definitely negative* and 4 to *definitely positive*), the sample average rating for the 40 initially cooperative children at the first visit would be 3 and would stay approximately constant through the phases of the ESBA (3 at the end of phase I and III and 2.9 at the end of phase II). Not surprisingly, for this cohort of children, according to the Stuart–Maxwell test, the differences between the distribution of the Frankl scale rating at the first visit and the corresponding distribution at the end of each phase were not statistically significant at the *α* = 0.05 level (the *p*-values for this test, one for each phase, were all greater than 0.17).

### 3.2 Findings of the proportional odds cumulative logit models

A total of 3,328 proportional odds cumulative logit models that relate the response variable (Frankl scale assessment) to a set of ten explanatory variables were fitted to the data. For baseline behavior, the binary variable *Init.Rating* (which takes the value 0 if the Frankl scale rating at the first visit is *negative* or *definitely negative* and 1 if the Frankl scale rating at the first visit is *positive*) was used to distinguish between the two cohorts of children with uncooperative and cooperative behaviors at the first visit. The other explanatory variables were *Phase* (indicating the phase at which the value of the response variable was retrospectively assessed), *Age*, *Gender*, medical history variables (*Type of delivery*, *Medications*, and *General anesthesi*a), and clinical variables (*Dental health status*, *Gingival status*, and *Observational plaque index*). The two variables *Nationality* and *Vaccines* were excluded from the modeling because of the large number of missing values and their low sample variability (as shown in Table 2, regardless of the cohort of children considered, more than 88.6% of the patients shared the same value for each variable).

Model selection was performed using the BIC criterion and the *1se rule* described previously. For the model selection, all 3,328 models were fitted using the same sample of 76 children, which corresponded to the largest sample for which complete information about the 10 regressors included in the modeling was available. The selected model (and the models of interest) was then reestimated using the largest sample for which complete information about the regressors was available. Parameter estimates, confidence intervals, and *p*-values for the coefficients of the full model, the minimum BIC model, and the best BIC-1se model are presented in Table 5. As shown in the table, four predictors (*Phase*, *Init.Rating*, *Medications*, and *Dental health status*) were statistically significant (at the level *α* = 0.05) in the full model. These four predictors also defined the model with minimum BIC.

**Table 5:** Maximum likelihood estimates for the coefficients of the full model, the best BIC model, and the best BIC-1se model.

Predictors	Cumulative logit models		
	Full model (*n* = 76) *β* (95% CI) [*p*-value]	Best BIC (*n* = 80) *β* (95% CI) [*p*-value]	Best BIC-1se (*n* = 85) *β* (95% CI) [*p*-value]
*Init.Rating*	2.72 (1.83, 3.75) [<0.001]	5.12 (3.25, 7.14) [<0.001]	2.48 (1.72, 3.36) [<0.001]
*Phase*	1.12 (0.68, 1.59) [<0.001]	1.43 (0.91, 1.99) [<0.001]	0.90 (0.51, 1.31) [<0.001]
*Medications (no)*	1.19 (0.36, 2.03) [0.005]	1.29 (0.51, 2.09) [0.001]	–
*Dental health status*	−0.18 (−0.34, −0.03) [0.018]	−0.21 (−0.33, −0.10) [<0.001]	–
*Age*	−0.21 NS	–	–
*Natural birth (yes)*	−0.08 NS	–	–
*Previous anesthesia (yes)*	0.64 NS	–	–
*Plaque 2*	−0.98 NS	–	–
*Plaque 3*	−1.14 NS	–	–
*Plaque 4*	−1.09 NS	–	–
*Gingival 2*	0.67 NS	–	–
*Gingival 3*	0.41 NS	–	–
Phase*Init.Rating (interaction)	–	−1.32 (−2.21, −0.45) [0.003]	–

For those coefficients that are statistically significant at *α* = 0.05 level, 95% confidence intervals (95% CI) and *p*-values are reported. Non-statistically significant coefficients are denoted by NS. Each model has been estimated using the largest sample for which complete information about the regressors in the model was available. The sample size (*n*) used to estimate the three models is reported in parenthesis next to the model's name.

The best BIC-1se model contained only the predictors *Phase* and *Init.Rating*, which emerged as the two most important explanatory variables in our statistical analysis. As shown in Table 5, both predictors were statistically significant and had positive estimated coefficients in the full model, the selected minimum BIC model, and the best BIC-1se model. Thus, this indicated that adding any of these two covariates to the model, improved the predictions of children’s behavior and cooperation as measured by the Frankl scale. This is confirmed by the corresponding 95% confidence intervals in the table that do not include the zero.

The positive estimated coefficient for *Init.Rating* indicated that children who were already cooperative at the beginning of the study (*Init.Rating* = 1) tended to have a more favorable Frankl scale assessment at the end of each of the three phases of the proposed protocol than those who were initially uncooperative (*Init.Rating* = 0). Similarly, the positive estimated coefficient for *Phase* indicated that children’s behavior and cooperation, as measured by the Frankl scale, tended to improve as they progressed from one phase to the next in the proposed approach (for a more technical interpretation of the coefficients of *Phase* and *Init.Rating* within the cumulative logit models in Table 5, see Appendix A).

**Figure 1:**
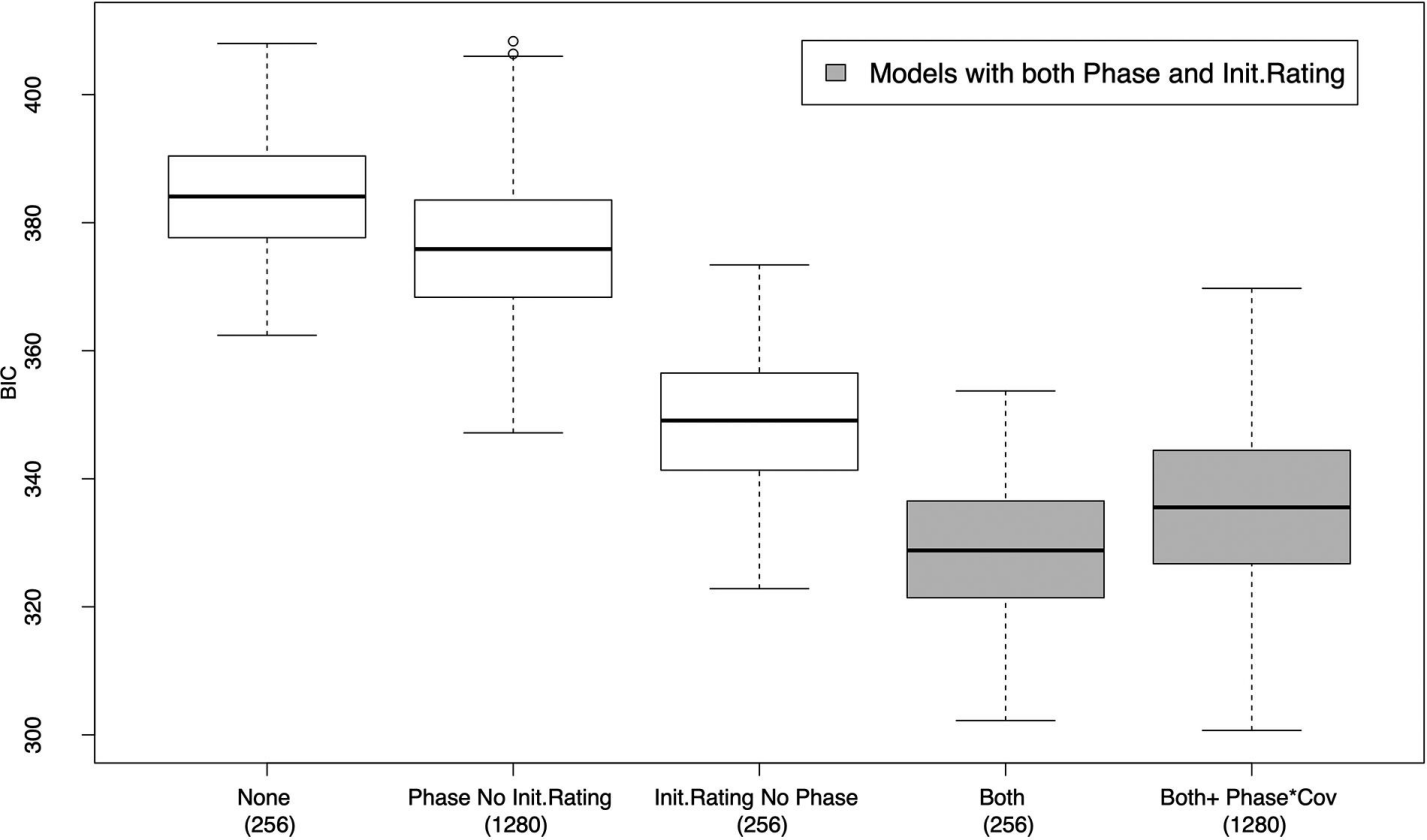
A side-by-side boxplot of BIC values for five different subgroups of the cumulative logit models considered in the analysis. For each group, the number of models in the group is reported in parenthesis below the group label*.* The boxplots of models that include both *Phase* and *Init.Rating* predictors are represented in gray. For the comparison of BIC values to be meaningful, all models were fitted using the same sample of 76 children, which corresponded to the largest sample for which complete information about the 10 regressors used in the modeling was available.

The importance of the *Phase* and *Init.Rating* variables is illustrated in the side-by-side boxplot of BIC scores in Figure 1. The figure considers a partition of the 3,328 cumulative logits models in five subgroups. For each subgroup, the number of models is reported in parenthesis below the subgroup label. The different subgroups correspond to (i) models that do not contain any of the two candidate predictors (*Phase* and *Init.Rating)*; (ii) models that contain *Phase* but not *Init.Rating*; (iii) models that contain *Init.Rating* but not *Phase*; (iv) models that contain both predictors (*Phase* and *Init.Rating*) but no interaction term; and (v) models with both *Phase* and *Init.Rating* and the interaction of *Phase* with another predictor.^1^

As shown in Figure 1, the corresponding BIC values cluster into four groups with increasing BIC range. The ordering of the four groups in terms of BIC makes apparent the relevance of *Phase* and *Init.Rating* as predictors of children’s behavior and cooperation as measured by the Frankl scale.

The best group (with BIC values in the range 300–369), represented in gray in Figure 1, comprised the models in subgroups (iv) and (v), which included both *Phase* and *Init.Rating*. The second-best group of models (with BIC values in the range 322–373) contained the *Init.Rating* predictor but not *Phase*. The third-best group (with BIC values in the range 347–408) contained *Phase* but not *Init.Rating*. The worst group of models (with BIC values in the range 362–407) consisted of models that did not contain either of the two regressors (neither *Init.Rating* nor *Phase*).

In line with this analysis, the best BIC-1se model contained only the covariates *Phase* and *Init.Rating*, both with positive and statistically significant coefficients. The estimated coefficients of *Phase* and *Init.rating* for the minimum BIC model were also positive and statistically significant. However, the presence of the interaction between *Phase* and *Init.rating* in the minimum BIC model, in practice, implied a different estimated coefficient for *Phase*, depending on the Frankl scale assessment at the first visit. Children with problematic behavior and limited cooperation at the beginning of the study (*Init.Rating* = 0) had an estimated coefficient of 1.43, which was more than 10 times larger than the corresponding coefficient for initially cooperative children at the beginning of the study (*Init.Rating* = 1), which was 0.11(1.43–1.32 = 0.11). Thus, in agreement with our descriptive findings, the minimum BIC model predicted that children with problematic behavior and limited cooperation at the beginning of the study tended to improve more across the different phases of the proposed approach compared with initially cooperative children. Despite the lack of an interaction term, the best BIC-1se model also captured this important feature of behavioral evolution during the ESBA program. As shown in Table 6, the best BIC-1se model predicted that an autistic patient uncooperative at the beginning of the study (*Init.Rating* = 0) with a probability of 0.40 will be cooperative by the end of phase I (Frankl scale at the end of phase I, *positive/definitely positive*). The probability of being cooperative had increased to 0.62 and 0.80 by phase II and phase III, respectively. In contrast, for an autistic patient already cooperative at the beginning of the study (*Init.Rating* = 1), these predicted probabilities of improvement were much lower and increased at a lower rate as a function of *Phase* (since there is little room for improvement). In particular, the best BIC-1se model predicted that an autistic patient cooperative at the beginning of the study with a probability of 0.02 (very small) would have improved their behavior and cooperation by the end of phase I (Frankl scale at the end of phase I, *definitely positive*). This probability increased only to 0.05 and 0.11 as the patients completed phase II and phase III, respectively.

**Table 6:** Predicted probabilities, based on the best-1se model, that an autistic child has improved by the end of phases I, II, and III.

Cohort of children	Predicted probability that a patient has improved by the end of		
	Phase 1	Phase 2	Phase 3
Uncooperative at first visit (*InitRating* = 0)	0.4021 (0.2822, 0.5251)	0.6227 (0.5322, 0.7107)	0.8019 (0.7066, 0.8834)
Cooperative at first visit (*InitRating* = 1)	0.0206 (0.0059, 0.0430)	0.0490 (0.0174, 0.0885)	0.1122 (0.0425, 0.1960)

According to our definition, a child initially uncooperative, i.e., with a Frankl scale rating of *negative* or *definitely negative* at first visit, has improved by the end of a certain phase, if their rating at the end of the phase is *positive* or *definitely positive*; a child initially cooperative, i.e., with a Frankl scale rating of *positive* at first visit, has improved by the end of a certain phase if their rating at the end of the phase is *definitely positive*. For each predicted probability, the corresponding 95% bootstrap-based confidence interval is shown in parenthesis.

The 95% confidence intervals in Table 6 provide informative bounds for the estimated probabilities of *improvement.* For example, with a 95% of confidence, it can be claimed that an autistic patient uncooperative at the beginning of the study with a probability of at least 0.28 will be cooperative by the end of phase I, and this probability will increase to 0.53 and 0.71 by the end of phases II and III, respectively. Similarly, with a 95% of confidence, it can be claimed that for an autistic patient cooperative at the beginning of the study, the probability of *improvement* by the end of phase III will be lower than 0.20.

**Figure 2:**
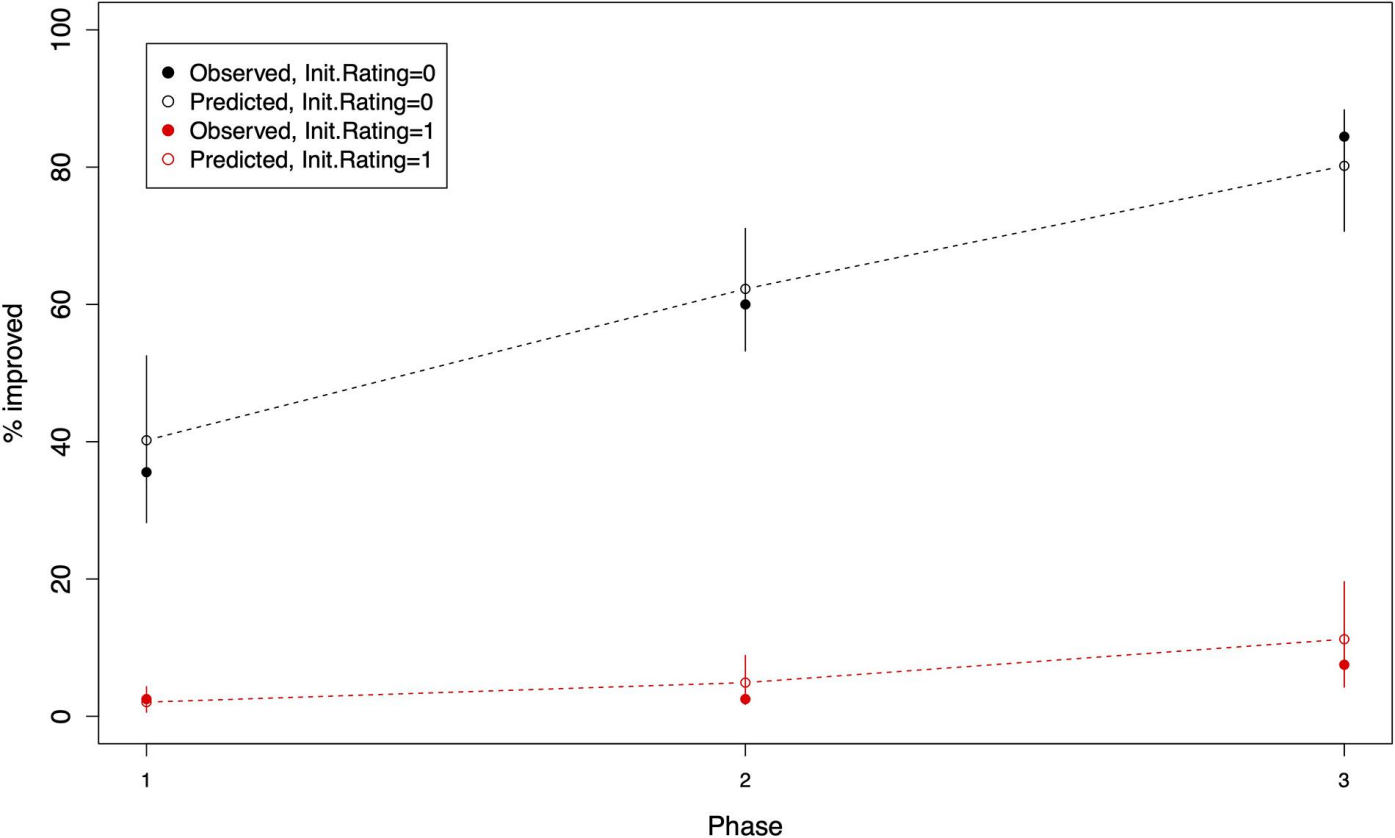
Observed and predicted percentages of children who improved their behavior with respect to the first visit, as a function of their initial rating and the phase of the protocol that they just completed. The black-filled circles represent the observed percentage of children with a negative initial rating (Frankl scale at first visit *negative* or *definitely negative*). The black empty circles represent the corresponding percentages predicted by the best BIC-1se model. 95% bootstrap-based confidence intervals are represented as vertical lines. The slope of the dashed line joining consecutive empty circles represents the rate at which the percentage of children who improve their behavior increases between one phase and the next according to the model's predicted probabilities. The corresponding line/points in red represents the same quantities but for the children with positive initial rating (Frankl scale at first visit *positive*).

The predicted probabilities in Table 6 can be transformed into predicted percentages multiplying each predicted probability by 100. These predicted percentages can be compared with the observed percentages obtained from Table 4 and can also be used as an indication of the fit of the best BIC-1se model to the data. This comparison is shown in Figure 2, which demonstrates that the best BIC-1se model quite effectively predicts the percentage of children who *improved* their behavior and cooperation across the different phases of the ESBA. The 95% bootstrap-based confidence intervals in each case contained the true proportion of children who *improved* their behavior. The good fit of the best BIC-1se model was confirmed by using the Pearson's chi-square test with an associated *p*-value of 0.148.

In order to test the proportional odds assumption underlying the best BIC-1se cumulative logit model, a likelihood ratio test (LRT) that examines the proportional odds model vs. the same model without proportional odds (i.e., vs. the model having a separate parameter for each logit) was conducted. The LRT test statistic was 7.64 with 4 degrees of freedom and a *p*-value of 0.11. Thus, there is no significant evidence to reject the proportional odds assumption at the *α* = 0.05 level.

## 4 Discussion

The ESBA approach first described in an earlier work (17) aims to increase cooperation in dental care treatment of children with ASD. This study represents the first attempt to quantify the effectiveness of this approach using data from two cohorts of children—initially uncooperative and initially cooperative—who completed the ESBA program at the Unit of Pediatric Dentistry, Policlinico Umberto I, Rome, between 2013 and 2020. The first cohort (uncooperative) contained 45 children who were rated as *negative* or *definitely negative* on the Frankl scale at their first visit. The second cohort (cooperative) contained 40 children who were rated as positive on the *Frankl* scale at their first visit. Although the study lacked a control group, the repeated-measures design allowed each subject to serve as their own control (32). The relevant outcome variables were related to the type of treatments performed and the *improvement* in the cooperation of the patient.

According to our definition, an initially uncooperative child was considered to have *improved* by the end of a given phase if the child's behavior had changed to cooperative (the Frankl scale changes from *negative* or *definitely negative* to *positive* or *definitely positive*). On the other hand, an initially cooperative child was considered to have *improved* by the end of a given phase if their Frankl rating had changed from *positive* to *definitely positive.* Our best BIC-1se cumulative logit model indicated a significant improvement across the different phases of the ESBA program in both cohorts of uncooperative and cooperative children, independent of the other explanatory variables in the study. With a 95% of confidence, we can claim that an autistic initially uncooperative child had at least 0.28 probability of improvement (i.e.*,* will be cooperative) by the end of phase I, with this increasing to 0.53 and 0.71 by the end of phases II and III, respectively. In contrast, the probabilities of improvement were much lower for initially cooperative children at the first visit. With a 95% of confidence, we can claim that an autistic initially cooperative child had a less than 0.20 probability of *improvement* (i.e., to experience a change in the Frankl scale rating from *positive* to *definitely positive*) by the end of phase III, and this probability could be as low as 0.04. These differences could be attributed to the different definitions of *improvement* in the two cohorts and the limited room for improvement among children already cooperative at the first visit.

The type of treatment variable is categorical with seven levels, five of which represent proper treatments (educational interventions, preventive procedures, dental cleaning, conservative procedures, and surgery procedures), while the other two refer to general anesthesia and check-up sessions. It was shown that, by the end of phase III, more than 64% of the 45 initially uncooperative children and more than 77.5% of the 40 initially cooperative children had received dental cleaning and preventive procedures. The percentages of initially uncooperative children who received conservative and surgical procedures also increased significantly during the ESBA program (amounting to more than 11% by the end of phase III). Despite this favorable evolution, these percentages remained much lower than (approximately one fifth and half) the corresponding percentages for the cohort of children who were initially cooperative.

In all phases, when educational intervention did not translate into collaboration, patients—if the urgency of the case required it—were referred for treatment under general anesthesia. Notably, the observed percentage of operations under anesthesia by the end of phase III of the ESBA (26.7% for the cohort of initially uncooperative children and 10% in the cohort of initially cooperative children) was quite low compared with the average number of operating room interventions required for this type of patient even for more banal treatments. In [12], it is reported that nearly four out of five (80%) autistic children required treatment under general anesthesia, and more than 60% needed a repeated general anesthesia session, underscoring the challenges in maintaining cooperation over time.

To the best of our knowledge, ESBA represents one of the most comprehensive attempts to establish a structured clinical–educational pathway for the dental management of children with autism spectrum disorder. The integration of educational techniques, a programmed schedule of appointments, adapted visual communication strategies, and long-term follow-up renders this model highly replicable and clearly oriented toward continuity of care. Related studies addressing dental management in children with ASD show marked variability in methodological rigor, outcome selection, and underlying conceptual frameworks. Sensory-adapted interventions, such as those described in ref. (8) and reviewed in ref. (10), typically assess short-term changes through physiological and behavioral indicators (heart rate, electrodermal activity, or standardized cooperation scores), capturing immediate responses to modifications of the clinical environment. These studies consistently reported significant reductions in physiological arousal and improvements in behavioral tolerance during treatment. However, these improvements were limited to the immediate session and did not extend to structured, sequential care plans. Educational approaches, including the video-modeling and communication-based protocols presented in refs. (33) and (34), rely on anticipatory learning strategies and evaluate outcomes through pre-/postbehavioral observations or cooperation scales. Both studies demonstrated meaningful increases in procedural acceptance. In one study (33), improvements in key cooperation behaviors were reported, while another (34) documented a decrease in refusal behaviors when the Picture Exchange Communication System (PECS) was used to support communication. Despite the effectiveness of these tools in facilitating short-term cooperation, none of these studies incorporated staged visit progression or examined the retention of behavioral gains over time. Hybrid models, such as those discussed in (7) and (14), combine elements of communication, desensitization, and sensory modulation. These approaches generally report broad improvements in patient tolerance. Although promising, these outcomes are derived from diverse and non-standardized metrics, and the interventions lack consistent guidance for visit sequencing or long-term follow-up, thus limiting their comparability with our results. In summary, existing studies are characterized by a pronounced heterogeneity of outcome measures. Sensory-based protocols depend largely on physiological and momentary behavioral markers, educational models rely on observational cooperation scores, and mixed approaches use composite but non-uniform indicators. Only a minority of studies incorporate clinically meaningful longitudinal endpoints (such as treatment completion or avoidance of general anesthesia), highlighting a gap in the standardization of outcomes. These inconsistencies restrict direct comparison with the ESBA and among studies and limit the feasibility of quantitative synthesis. Developing a core outcome set for ASD-focused dental research would support methodological harmonization, improve comparability, and strengthen the evidence base.

It is important to acknowledge some limitations of the results and statistical analyses reported in this study. Although the Unit of Pediatric Dentistry at Policlinico Umberto I in Rome, where the sample was collected, attends children with autism without targeting any special subgroup, sampling bias might still be present. Since participation in the study was voluntary, children with autism from families with higher parental education and socioeconomic status may have been more likely to participate due to the family's awareness of the importance of such studies and the ability to attend visits regularly to complete the program. In addition, despite the fact that the male-to-female ratio in our sample was approximately 3:1, which is in agreement with the general prevalence ratio of autism reported by the WHO (6), and in line with previous works on the subject (5, 6, 11, 20, 35), the clinical data collected in this study also suggested poor oral health in the ASD population, the sample used (with *n* = 85 ASD children aged between 7 and 12 years) might not be representative of the ASD population of this age range. As a result, the percentages of the type of treatment performed by phase and the percentage of children who *improved* their behavior in the two cohorts as presented in Tables 3, 4, and 6 may have limited generalization. These issues are related to a second limitation, which is the exclusion in the study of some potentially relevant predictors such as parental education and occupation, autism severity and support needs, history of dental visits (prior to the ESBA program), and comorbidities. Numerous studies have shown that ASD is commonly associated with a broad range of comorbidities, including neurological, gastrointestinal, immunological, and psychiatric conditions, which can affect clinical management and procedural planning. These comorbidities are not only highly prevalent, but also heterogeneous in their expression and etiology, often requiring tailored approaches to care (36, 37). The limited sample size might have also prevented us from finding other statistically significant predictors.

An additional limitation is potential measurement bias. Although the Frankl scale rating system is behaviorally defined, these definitions still maintain a certain degree of subjectivity that translates into a potential risk for operator-dependent rating of children’s behavior and therefore measurement bias (38). This can be especially true in our study, since the Frankl scale was assessed retrospectively based on the annotations of the children’s behaviors in the clinical charts, and because for children with ASD, neurodivergent behaviors may be interpreted inconsistently across clinicians (39, 40). On the other hand, we believe that the training program of the dentists participating in the ESBA—who must complete a 1-year training period with a qualified autism therapist—and the structured methodology of the ESBA—which includes scheduled sequential visits, standardized communication strategies, and longitudinal clinical charts—may mitigate these problems. Ratings at the end of each phase of the program were based on repeated, comparable observations, reducing the reliance on single-visit impressions. Future modifications to current clinical charts that enhance standardization and reduce subjectivity in the assessment of child behavior and cooperation will greatly benefit an assessment of the effectiveness of the program in future studies. Finally, although reduction of dental anxiety was not the primary outcome of our study (since anxiety was relevant to the extent that it limited cooperation), the absence of anxiety records for the 85 children in our sample represents another limitation. Inclusion of some measure of anxiety, for example the Venham anxiety rating scale (38), would allow a more comprehensive assessment of the effectiveness of the ESBA and could be used to define more specific inclusion criteria in future studies (in which assessment of the ESBA could focus on cohorts of children with specific ASD diagnosis, comorbidities, dental anxiety, and age range).

As stated at the beginning of the study, this in an ongoing project. We expect to address the pending and other potential issues in future work.

## 5 Conclusions

The dental care of autistic children can present challenges because of their over-responsivity to environmental stressors and their lower communication ability, which significantly reduce the efficacy of conventional behavioral management strategies. These often result in uncooperative behavior and necessitate the use of general anesthesia, a solution that constitutes a medical risk for the patient, limits the number and type of interventions that can be performed, and is particularly problematic given the poor oral hygiene status that often characterizes children with ASD. Educational approaches, that position education as a tool for perfecting dental treatment by limiting traumatic experiences to the patient and allowing continuity in the procedures so that cooperation can be improved and the use of general anesthesia can be reduced, offer a promising avenue to address the aforementioned challenges. Their effectiveness, however, should be tested and quantified.

This study represents the first attempt to assess the effectiveness of the ESBA, implemented at the Unit of Pediatric Dentistry of Policlinico Umberto I in Rome (Italy). According to our best cumulative logit model, (i) the predicted percentage of autistic children initially uncooperative who will become cooperative (i.e., will change their Frankl rating to *positive* or *definitely positive*) by the end of the ESBA program is 80; (ii) the predicted percentage of autistic children already cooperative at the first visit (Frankl scale at first visit *positive*) who will improve their level of cooperation (i.e., will change their Frankl rating to *definitely positive*) by the end of the program is 11.

In addition, we demonstrated that the percentage of initially cooperative and uncooperative children on whom it is possible to carry out a series of dental interventions without resorting to general anesthesia significantly increases across the phases of the ESBA program.

Our study contributes to the growing body of evidence supporting the efficacy of multidisciplinary approaches, such as the ESBA program, in improving oral health outcomes and enhancing patient experiences among children with ASD, thereby reducing the need for invasive treatments.

The findings from this study can inform clinical practice in pediatric dentistry, particularly in managing patients with ASD. We hope that the promising results reported in this study will motivate researchers and provide a starting point for other groups and medical teams to implant and implement similar or alternative educational approaches. The sharing of results and experiences of these different groups will improve our understanding of the strengths and limitations of the individual approaches and will be of great help in defining new and better strategies for oral care of ASD children.

## Data Availability

The raw data supporting the conclusions of this article will be made available by the authors without undue reservation.

## Footnotes

^1^Note that subgroups (i), (iii), and (iv) contain models without interaction terms, while group (v) only contains models with interactions. On the other hand, subgroup (ii) is mixed. It comprises both models with no interaction term (that do not contain *Init.Rating*) and models with the interaction of *Phase* with another of the remaining eight explanatory variables other than *Init.Rating* (and that do not contain *Init.Rating*).

## Appendix A

The best BIC-1se model is a cumulative logit model with proportional odds, which contains only two predictors, *Phase* and *Init.Rating*. The model is parametrized as follows:

$$\begin{array}{r} \mathrm{logit}[P(\mathrm{Frankl}\;\mathrm{scale}\;\leq j)]=\alpha_{j}-\beta_{1}\cdot (Init.Rating)-\beta_{2}\cdot (Phase)+\xi ,\;\;j=1,2,3 \end{array}$$

In this formulation, ξ is an error term, *Phase* is considered a quantitative variable that takes the value of 1, 2, or 3 depending on whether the Frankl scale assessment has been made at the end of phase I, II, or III, respectively, and the ordered levels of the Frankl scale have been codified with the integers 1, 2, 3, or 4 (1 corresponding to *definitely negative* and 4 to *definitely positive*). The model contains five parameters: three intercepts $\alpha_{j}^{\prime }s,$ which are of no interest except for computing the response probabilities, and two main-effects parameters, $\beta_{1}$ and $\beta_{2}$, which quantify the effect of the explanatory variables *Init.Rating* and *Phase* on the distribution of the response variable (Frankl scale assessment).

In the best BIC-1se model, an increase in phase is significantly associated with an increase in the Frankl scale [*β*_2_ = 0.90, 95% CI (0.51, 1.31), *p*-value < 0.001]. In particular, for phase *k* *+* *1*, the estimated odds of the Frankl scale ≥ *j* (instead of <j) is 2.46 (e^0.90^) times the estimated odds for phase *k* (*k* = 1,2; *j* = 1,2,3). Similarly, for any given phase of the program, children who are more cooperative at the beginning of the study tend to have higher ratings in Frankl scale than those with more problematic behavior [*β*_1_ = 2.48, 95% CI (1.85, 3.58), *p*-value<0.001]. In particular, the estimated odds of the Frankl scale ≥ *j* (instead of <*j*) for an autistic patient who is cooperative at the beginning of the study (*Init.Rating* = 1) is 11.94 (e^2.48^) times the estimated odds for an autistic patient who is uncooperative at the beginning of the study (*Init.Rating* = 0).
